# Is oxygen therapy beneficial for normoxemic patients with acute heart failure? A propensity score matched study

**DOI:** 10.1186/s40779-021-00330-7

**Published:** 2021-07-09

**Authors:** Yue Yu, Ren-Qi Yao, Yu-Feng Zhang, Su-Yu Wang, Wang Xi, Jun-Nan Wang, Xiao-Yi Huang, Yong-Ming Yao, Zhi-Nong Wang

**Affiliations:** 1 Department of Cardiothoracic Surgery, Changzheng Hospital, Naval Medical University, 415 Fengyang Road, Huangpu District, Shanghai, 200003 China; 2 Trauma Research Center, Fourth Medical Center and Medical Innovation Research Department of the Chinese PLA General Hospital, 51 Fucheng Road, Haidian District, Beijing, 100048 China; 3 Department of Burn Surgery, Changhai Hospital, Naval Medical University, Shanghai, 200433 China; 4 Medical Research Center of War Injuries and Trauma, Changzheng Hospital, Naval Medical University, Shanghai, 200003 China; 5 Department of Pathology, Changhai Hospital, Naval Medical University, Shanghai, 200433 China

**Keywords:** Acute heart failure, Death, Hyperoxia, Mortality, Oxygen therapy

## Abstract

**Background:**

The clinical efficiency of routine oxygen therapy is uncertain in patients with acute heart failure (AHF) who do not have hypoxemia. The aim of this study was to investigate the association between oxygen therapy and clinical outcomes in normoxemic patients hospitalized with AHF using real-world data.

**Methods:**

Normoxemic patients diagnosed with AHF on ICU admission from the electronic ICU (eICU) Collaborative Research Database were included in the current study, in which the study population was divided into the oxygen therapy group and the ambient-air group. Propensity score matching (PSM) was applied to create a balanced covariate distribution between patients receiving supplemental oxygen and those exposed to ambient air. Linear regression and logistic regression models were performed to assess the associations between oxygen therapy and length of stay (LOS), and all-cause in-hospital as well as ICU mortality rates, respectively. A series of sensitivity and subgroup analyses were conducted to further validate the robustness of our findings.

**Results:**

A total of 2922 normoxemic patients with AHF were finally included in the analysis. Overall, 42.1% (1230/2922) patients were exposed to oxygen therapy, and 57.9% (1692/2922) patients did not receive oxygen therapy (defined as the ambient-air group). After PSM analysis, 1122 pairs of patients were matched: each patient receiving oxygen therapy was matched with a patient without receiving supplemental oxygen. The multivariable logistic model showed that there was no significant interaction between the ambient air and oxygen group for all-cause in-hospital mortality [odds ratio (*OR*) 1.30; 95% confidence interval (CI) 0.92–1.82; *P =* 0.138] or ICU mortality (*OR* 1.39; 95% CI 0.83–2.32; *P =* 0.206) in the post-PSM cohorts. In addition, linear regression analysis revealed that oxygen therapy was associated with prolonged ICU LOS (*OR* 1.11; 95% CI 1.06–1.15; *P* <  0.001) and hospital LOS (*OR* 1.06; 95% CI 1.01–1.10; *P =* 0.009) after PSM. Furthermore, the absence of an effect of supplemental oxygen on mortality was consistent in all subgroups.

**Conclusion:**

Routine use of supplemental oxygen in AHF patients without hypoxemia was not found to reduce all-cause in-hospital mortality or ICU mortality.

**Supplementary Information:**

The online version contains supplementary material available at 10.1186/s40779-021-00330-7.

## Background

Acute heart failure (AHF) is a life-threatening medical condition requiring immediate treatment and leading to urgent hospitalization [1]. Supplemental oxygen therapy is a routine treatment modality in the management of AHF patients [2, 3]. The underlying rationale behind oxygen therapy for AHF patients is to relieve dyspnea, or improve oxygenation for the threatened myocardial tissue, thereby alleviating myocardial injury and improving cardiac function [4–6]. However, above-normal arterial oxygen tension can cause systemic vasoconstriction [7], overproduction of reactive oxygen species (ROS) [8], and, consequently, worsening of heart failure (HF). Currently, most studies have focused solely on AHF patients with refractory and progressive hypoxemia, and oxygen therapy is recommended in patients with pulse oximetry-derived oxygen saturation (SpO_2_) < 90% to correct hypoxemia [2, 9]. For normoxemic patients (defined as SpO_2_ between 90 and 100%) presenting with AHF, whether supplementary oxygen provides benefit or not remains highly uncertain.

This topic has been studied in other non-AHF clinical settings. For example, several randomized controlled trials (RCTs) have demonstrated that supplemental oxygen had no clinical benefits among patients without hypoxemia presenting with acute myocardial infarction (AMI) and others have suggested possible harm [10–15]. Considering the contradictory findings regarding supplemental oxygen therapy, recent HF guidelines diverge from the previous consensus that oxygen should be administered routinely among AHF patients irrespective of oxygen saturation at baseline [16–18]. However, this new direction is solely based on expert opinion or low-quality and nonrandomized clinical trials with a small sample size [6, 19, 20]. Given the cost of oxygen therapy and the ubiquitous use of oxygen among patients hospitalized with AHF, it is necessary to evaluate the correlation between supplemental oxygen and clinical outcomes [4, 21, 22]. Thus, this present study examined the hypothesis that routine use of supplemental oxygen in normoxemic AHF patients was not found to reduce all-cause in-hospital mortality or ICU mortality.

## Methods

### Data sources and ethical statement

The electronic Intensive Care Unit (eICU) Collaborative Research Database was a multi-center ICU database for more than 200,000 admissions from over 200 hospitals across the USA between 2014 and 2015 [23]. The eICU database documents contained comprehensive charted events, including demographic data, diagnosis information via International Classification of Diseases, Ninth Revision (ICD-9) codes, vital sign measurements, laboratory findings and blood gas analyses, hourly physiologic readings from bedside monitors, various scoring systems, treatment information, and clinical outcomes. The establishment of the eICU database was approved by the Institutional Review Boards of the Massachusetts Institute of Technology (Cambridge, MA, USA). All the data were made anonymous prior to research analyses by the eICU programme, and hence the requirement for informed consent was waived. The study complied with the ethical standards laid down in the 1964 Declaration of Helsinki and its later amendments. We finished the “Protecting Human Research Participants” curriculum, and obtained permission to access the dataset (authorization code: 33281932). In addition, we conducted this study in accordance with the STrengthening the Reporting of OBservational studies in Epidemiology (STROBE) statement [24].

### Population selection

We included all ICU patients (aged > 30 years) with a primary diagnosis of AHF using ICD-9 diagnosis codes (ICD-9 codes: 404.91, 415.0, 428.0, 428.1, 428.21, 428.23, 428.31, 428.33, 428.41, and 428.43) from the eICU database. Patients were excluded who (1) had SpO_2_ < 90% on admission; (2) presented with cardiac arrest or cardiogenic shock on admission; (3) were at risk of oxygen-induced hypercapnia (chronic obstructive pulmonary disease, asthma, or pneumonia) on admission; (4) stayed in the ICU for less than 24 h; (5) required more intensive oxygen therapy including non-invasive ventilation (NIV) or invasive ventilation through endotracheal intubation during hospitalization; (6) received oxygen therapy at a flow rate of 10 L/min or more (10 L/min is accepted as the maximum threshold value of flow rate for using face mask or nasal cannula); and (7) had incomplete or unobtainable documented information about oxygen saturation, oxygen therapy, and clinical outcomes.

### Data extraction and data processing

The data were extracted from the database using structured query language (SQL) with PostgreSQL (version 9.6). The code that supported the eICU documentation and website was publicly available (https://github.com/mit-lcp/eicu-code). Demographic information included age, gender, and body mass index (BMI). BMI was calculated as weight (kg) divided by height^2^ (m^2^), using height and weight reported at the time of admission. Comorbidities on admission included sepsis, acute renal injury, and acute coronary syndrome. History of disease included atrial fibrillation, coronary artery disease (CAS), hypertension, stroke, diabetes mellitus, chronic kidney disease, and hyperthyroidism. Vital signs at presentation included systolic blood pressure, heart rate, and SpO_2_ on the first day. Laboratory findings and blood gas analysis data included albumin, creatinine, glucose, blood urea nitrogen, hematocrit, hemoglobin, blood platelets, white blood cells, potassium, and sodium. If vital signs were measured multiple times or patients received a laboratory test more than once during their hospitalization, an initial data on the first day after ICU admission was extracted for subsequent analyses. The severity of illness was assessed by three scoring systems [the Oxford Acute Severity of Illness Score (OASIS), the Sequential Organ Failure Assessment score (SOFA), and the Glasgow Coma Scale (GCS)]. These scoring systems were calculated within the first 24 h after admission using the values associated with the greatest severity of illness.

For treatment information, each admitted patient was treated independently, although some patients in the dataset might have had multiple admissions. Routine oxygen therapy in this study could refer to oxygen supplementation methods either via face mask or nasal cannula, because the eICU database did not provide detailed information to differentiate these two methods. Patients who received NIV or invasive ventilation during hospitalization were excluded. We took the average value of the SpO_2_ measurements during oxygen therapy as a measure of the central tendency of oxygen exposure. To address concerns about the time dependency of oxygen exposure, the duration of oxygen therapy was also recorded for subsequent analyses. Other treatment information included intra-aortic balloon pump, renal replacement treatment, and in-hospital medication administration [inotrope, diuretic, angiotensin-converting-enzyme inhibitors/angiotensin receptor blockers (ACEI/ARB), calcium channel blocker (CCB), and beta-blocker]. Additionally, all the therapeutic methods (intra-aortic balloon pump, renal replacement treatment, and in-hospital medication) were implemented in all study participants prior to the initiation of oxygen therapy.

As severe data missing might lead to bias, variables with over 20% missing values were not taken into subsequent analysis. Correspondingly, multiple imputation was used for processing variables with less than 20% missing values [25, 26].

### Endpoints

The primary endpoint of our study was all-cause in-hospital mortality, which was defined as survival status at hospital discharge. We selected all-cause ICU mortality and ICU and hospital length of stay (LOS) as secondary endpoints. ICU and hospital LOS were calculated as the total duration spent in the ICU and hospital since hospital admission separately. Patients with missing survival outcome information were excluded from the final cohort.

### Statistical analysis

Normoxemic patients with AHF were divided into the oxygen therapy group and the ambient-air group. If the measurement data were normally distributed and the variance was homogeneous, data were characterized as mean ± standardized differences (SD) and compared between groups using a Student’s *t* test. If the requirements were not satisfied, data were represented by median [interquartile range (IQR)], and the Kruskal Wallis rank test was used for comparisons between groups. Numeration data were expressed as absolute values and percentages; the Pearson’s χ^2^ test or Fisher’s exact test was chosen for statistical analyses as appropriate.

Given the observational nature of the current study, propensity score matching (PSM) was used to minimize the effect of potential confounders. A logistic regression model was constructed to calculate and assign each patient a propensity score, which was defined as the likelihood of being exposed to an intervention. Next, 1:1 matching (the oxygen therapy group vs. the ambient-air group) without replacement was performed using a nearest neighbor matching algorithm, with a fixed caliper width of 0.05. The standardized mean difference (SMD) was calculated to evaluate the efficiency of PSM in reducing the differences between the two groups.

In the pre-PSM and post-PSM cohorts, logistic regression models were employed to investigate associations between oxygen therapy and clinical outcomes adjusting for confounding variables selected based on *P* <  0.05 in the univariate analysis, in which the Akaike information criterion was applied as the selection criteria of the optimal model. Linear regression was used to assess the correlation of oxygen therapy with length of stay, and the odds ratios (*OR*s) were presented using the formula *OR* = e^βi^. A series of subgroup analyses were performed to further assess the association between oxygen therapy and all-cause in-hospital mortality, including duration of oxygen therapy, median SpO_2_ during hospitalization, age, De Nova AHF, history of atrial fibrillation, history of myocardial infarction, history of stroke, history of hypertension, history of chronic kidney disease, and renal replacement treatment. In the subgroup analyses of patients’ duration of oxygen therapy, the reference group was defined as all the patients of the ambient-air group. In the other subgroup analyses, the reference group was defined as the patients of the ambient-air group in the corresponding subgroup.

A two-tailed *P* value of less than 0.050 was considered to be statistical significance. All statistical analyses were performed using SPSS software (version 22.0; IBM Corporation, St. Louis, Missouri, USA) and R software (version.3.6.1,The R Project for Statistical Computing, TX, USA; http://www.r-project.org).

## Results

### Basic characteristics

**Fig. 1:**
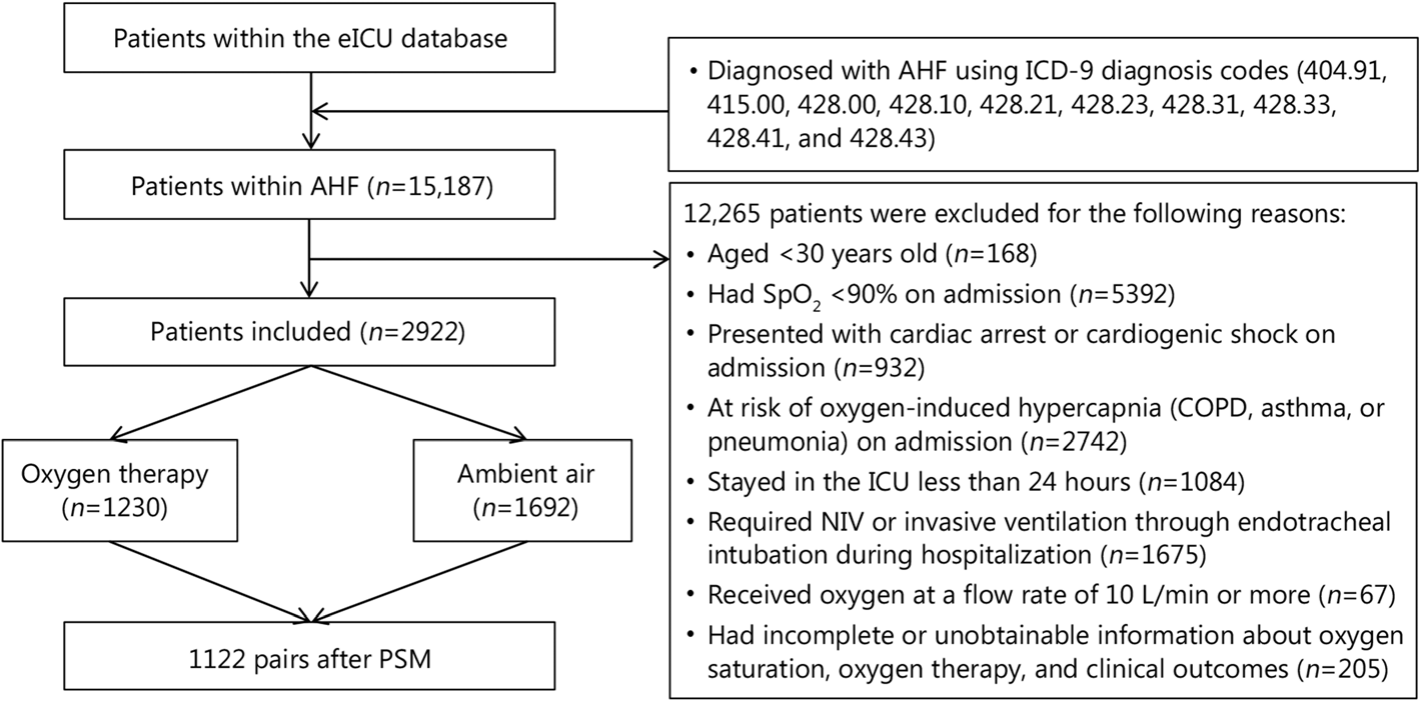
Case inclusion flowchart. eICU electronic intensive care unit, AHF acute heart failure, PSM propensity score matching, ICD-9 international classification of diseases ninth revision, SpO2 pulse oximetry-derived oxygen saturation, COPD chronic obstructive pulmonary disease, NIV non-invasive ventilation

During the study period, 15,187 critically ill patients were admitted with AHF. Consequently, after excluding the patients according to the selection criteria, 2922 eligible patients were enrolled in the current study (Fig. 1). The patients included in the final cohort had a median age of 72 (61–82) years, 54.3% (1586/2922) patients were men, and 39.5% (1155/2922) patients were identified as having de novo AHF (Table 1).

**Table 1:** Baseline characteristics of normoxemic patients with AHF between the two groups before matching

Characteristics^a^	Total (***n =*** 2922)	Ambient Air (***n =*** 1692)	Oxygen Therapy (***n =*** 1230)	***P*** value	SMD
Demographics					
Age (years)	72 (61–82)	72 (61–82)	72 (60–82)	0.316	0.047
Gender [male, *n* (%)]	1586 (54.3)	915 (54.1)	671 (54.6)	0.799	0.010
BMI (kg/m^2^)	28.7 (24.2–34.5)	28.6 (24.3–34.4)	28.8 (24.2–34.7)	0.716	0.004
de novo AHF [*n* (%)]	1155 (39.5)	659 (38.9)	496 (40.3)	0.452	0.028
Comorbidities on admission [*n* (%)]					
Sepsis	306 (10.5)	183 (10.8)	123 (10.0)	0.477	0.027
AKI	65 (2.2%)	45 (2.7%)	20 (1.6%)	0.061	0.071
History of disease [*n* (%)]					
AF	668 (22.9)	381 (22.5)	287 (23.3)	0.604	0.019
CAD					
MI	561 (19.2)	331 (19.6)	230 (18.7)	0.559	0.022
PCI	335 (11.5)	200 (11.8)	135 (11.0)	0.479	0.027
CABG	344 (11.8)	194 (11.5)	150 (12.2)	0.546	0.023
Hypertension	1869 (64.0)	1041 (61.5)	828 (67.3)	0.001	0.121
Stroke	301 (10.3)	178 (10.5)	123 (10.0)	0.648	0.017
DM	1145 (39.2)	656 (38.8)	489 (39.8)	0.590	0.020
CKD	775 (26.5)	451 (26.7)	324 (26.3)	0.850	0.007
Hyperthyroidism	346 (11.8)	190 (11.2)	156 (12.7)	0.230	0.045
Vital signs at presentation					
SBP (mmHg)	121.0 (120.0–127.0)	121.0 (120.0–127.0)	121.0 (120.0–127.0)	0.467	0.021
HR (beats/min)	86.0 (73.0–101.0)	86.0 (73.0–101.0)	87.0 (74.0–101.0)	0.179	0.043
SpO_2_ (%)	97.0 (95.0–99.0)	97.0 (95.0–99.0)	97.0 (95.0–99.0)	0.946	0.013
Laboratory findings and blood gas analysis					
Albumin (mg/dL)	3.1 (2.7–3.5)	3.1 (2.7–3.5)	3.1 (2.7–3.5)	0.941	0.007
Creatinine (μmol/L)	1.3 (0.9–2.2)	1.310 (0.9–2.2)	1.3 (0.9–2.1)	0.195	0.062
Glucose (mg/dL)	109.0 (91.0–138.0)	109.0 (91.0–137.3)	110.0 (91.0–139.0)	0.420	0.010
BUN (mg/dL)	27.0 (18.0–44.0)	27.0 (18.0–44.0)	27.0 (18.0–43.0)	0.957	0.038
Hematocrit (%)	32.1 (6.9)	31.9 (6.9)	32.3 (6.9)	0.110	0.060
Hemoglobin (g/dL)	10.5 (8.8–12.1)	10.5 (8.8–12.0)	10.5 (8.9–12.2)	0.137	0.062
Platelet (10^9^/L)	183.5 (136.0–241.0)	180.0 (134.0–238.0)	186.8 (138.0–244.0)	0.072	0.101
WBC (10^9^/L)	8.5 (6.3–11.6)	8.4 (6.3–11.4)	8.6 (6.4–11.8)	0.224	0.011
Potassium (mmol/L)	3.9 (3.5–4.3)	3.9 (3.5–4.3)	3.9 (3.5–4.3)	0.784	0.018
Sodium (mmol/L)	136.0 (133.0–139.0)	137.0 (134.0–140.0)	135.0 (132.0–138.0)	0.002	0.125
Scoring system					
OASIS	19.0 (14.0–25.0)	20.0 (15.0–26.0)	19.0 (12.0–25.0)	< 0.001	0.214
SOFA	4.0 (2.0–6.0)	4.0 (2.0–6.0)	4.0 (2.0–6.0)	0.248	0.053
GCS	15.0 (15.0–15.0)	15.0 (15.0–15.0)	15.0 (15.0–15.0)	0.665	0.008
Management of AHF [*n* (%)]					
Intra-aortic balloon pump	41 (1.4)	23 (1.4)	18 (1.5)	0.813	0.009
RRT	149 (5.1)	102 (6.0)	47 (3.8)	0.007	0.102
In-hospital medication					
Inotrope	465 (15.9)	242 (14.3)	223 (18.1)	0.001	0.158
Diuretic	2221 (76.0)	1259 (74.4)	962 (78.2)	< 0.001	0.150
ACEI/ARB	1730 (59.2)	958 (56.6)	772 (62.8)	< 0.001	0.214
CCB	171 (5.9)	82 (4.8)	89 (7.2)	0.007	0.100
Beta-blocker	1495 (51.2)	690 (40.8)	805 (65.4)	< 0.001	0.371
Oxygen therapy					
Duration of oxygen therapy (days)	/	/	2.5 (1.6–4.1)	/	/
Median SpO_2_ during treatment period (%)	97.9 (96.5–98.8)	97.1 (95.5–97.8)	98.7 (97.4–99.8)	< 0.001	/

^a^Values are *n* (%) or median (interquartile range). *SMD* standardized mean difference, *BMI* body mass index, *AHF* acute heart failure, *AKI* acute kidney injury, *AF* atrial fibrillation, *CAD* coronary artery disease, *MI* myocardial infarction, *PCI* percutaneous coronary intervention, *CABG* coronary artery bypass grafting, *DM* diabetes mellitus, *CKD* chronic kidney disease, *SBP* systolic blood pressure, *HR* heart rate, *SpO*_*2*_ pulse oximetry-derived oxygen saturation, *BUN* blood urea nitrogen, *WBC* white blood cell, *OASIS* oxford acute severity of illness score, *SOFA* sequential organ failure assessment score, *GCS* glasgow coma scale, *RRT* renal replacement treatment, *ACEI/ARB* angiotensin-converting-enzyme inhibitors/angiotensin receptor blockers, *CCB* calcium channel blocker

Overall, 42.1% (1230/2922) patients were exposed to oxygen therapy, and 57.9% (1692/2922) patients did not receive oxygen therapy (defined as the ambient-air group). The median duration of oxygen therapy was 2.5 (1.6–4.1) days. The comparison of baseline characteristics between these two groups was summarized in Table 1. There were significant differences in history of disease [hypertension: 61.5% (1041/1692) vs. 67.3% (828/1230); *P =* 0.001] and scoring systems [OASIS: 20.0 (15.0–26.0) vs. 19.0 (12.0–25.0); *P* <  0.001] between the ambient-air group and the oxygen therapy group. Additionally, patients exposed to oxygen therapy were more likely to receive medication administration during hospitalization [inotropes: 14.3% (242/1692) vs. 18.1% (223/1230); *P =* 0.001; diuretic: 74.4% (1259/1692) vs. 78.2% (962/1230); *P* <  0.001; ACEI/ARB: 56.6% (958/1692) vs. 62.8% (772/1230); *P* <  0.001; CCB: 4.8% (82/1692) vs. 7.2% (89/1230); *P =* 0.007; and beta-blocker: 40.8% (690/1692) vs. 65.4% (805/1230); *P* <  0.001]. No difference was observed in admission SpO_2_ level [97.0% (95.0–99.0%) vs. 97.0% (95.0–99.0%); *P =* 0.946] between these two groups. Furthermore, during the intervention, mean SpO_2_ level of the oxygen therapy group was higher than that of the ambient-air group [98.7% (97.4–99.8%) vs. 97.1% (95.5–97.8%); *P* <  0.001].

### Relationship between oxygen therapy and outcomes

Univariable and multivariable logistic regression models were employed to examine the difference in all-cause in-hospital and ICU mortality rates between these two groups. In the pre-matched cohort, 6.1% (104/1692) patients in the ambient-air arm and 8.0% (98/1230) patients in the oxygen therapy arm died in the hospital (*OR* 1.32; 95% confidence interval [CI] 0.99–1.76; *P =* 0.056) (Table 2; Table S1). 3.5% (43/1230) patients in the oxygen group died in the ICU in comparison to 2.5% (42/1692) patients in the ambient-air group (*OR* 1.42; 95% CI 0.92–2.19; *P =* 0.109) (Table 2; Table S2). By the multivariable analysis, no differences were observed for in-hospital mortality (*OR* 1.34; 95% CI 0.98–1.84; *P =* 0.067) (Table 2; Table S1) or ICU mortality (*OR* 1.58; 95% CI 0.97–2.56; *P =* 0.066) after adjusting for possible confounding factors associated with mortality (Table 2; Table S1; Table S2). Moreover, linear regression was used to evaluate the association between oxygen therapy and ICU as well as hospital LOS. Nevertheless, oxygen therapy was significantly associated with prolonged ICU LOS (*OR* 1.15; 95% CI 1.11–1.19; *P* <  0.001) and hospital LOS (*OR* 1.07; 95% CI 1.04–1.11; *P* <  0.001) (Table 2).

**Table 2:** Association between oxygen therapy and clinical outcomes in normoxemic patients with AHF

Clinical outcomes^a^	Ambient Air	Oxygen Therapy	***OR*** (95% CI)	***P*** value
Pre-matched cohort	*n =* 1692	*n =* 1230		
All-cause in-hospital mortality [*n* (%)]				
Univariable logistic model	104 (6.1)	98 (8.0)	1.32 (0.99–1.76)	0.056
Multivariable logistic model	104 (6.1)	98 (8.0)	1.34 (0.98–1.84)	0.067
All-cause ICU mortality [*n* (%)]				
Univariable logistic model	42 (2.5)	43 (3.5)	1.42 (0.92–2.19)	0.109
Multivariable logistic model	42 (2.5)	43 (3.5)	1.58 (0.97–2.56)	0.066
Length of ICU stay (days)	2.2 (1.5–3.5)	2.7 (1.8–4.6)	1.15 (1.11–1.19)	< 0.001
Length of hospital stay (days)	6.5 (4.0–11.3)	7.8 (4.8–13.1)	1.07 (1.04–1.11)	< 0.001
Post-matched cohort	*n =* 1122	*n =* 1122		
All-cause in-hospital mortality [*n* (%)]				
Univariable logistic model	74 (6.6)	89 (7.9)	1.22 (0.89–1.68)	0.223
Multivariable logistic model	74 (6.6)	89 (7.9)	1.30 (0.92–1.82)	0.138
All-cause ICU mortality [*n* (%)]				
Univariable logistic model	34 (3.0)	40 (3.6)	1.18 (0.74–1.88)	0.479
Multivariable logistic model	34 (3.0)	40 (3.6)	1.39 (0.83–2.32)	0.206
Length of ICU stay (days)	2.2 (1.5–3.8)	2.7 (1.7–4.4)	1.11 (1.06–1.15)	< 0.001
Length of hospital stay (days)	6.8 (4.1–11.2)	7.6 (4.6–12.9)	1.06 (1.01–1.10)	0.009

^a^Values are *n* (%) or median (interquartile range). *AHF* acute heart failure, *ICU* intensive care unit, *OR* odds ratio, *CI* confidence interval

By the PSM analysis, 2244 patients remained in the post-PSM cohort, and 1122 patients who received oxygen therapy were matched with 1122 patients who did not receive oxygen. All the variables listed in Table 1 (except for duration of oxygen therapy and mean SpO_2_ during treatment period) were considered in the PSM analysis. The characteristics of matched patients were compared, and the SMDs for all the individual covariates were provided. Differences in all variables between the oxygen therapy group and the ambient-air group were reduced and had no statistical significances measured by SMDs that were less than 10% for all variables (Table 1; Table S3).

Similar to the results in the pre-matched model, the logistic model showed that there was no significant interaction between the ambient air and oxygen groups with regard to all-cause in-hospital mortality (univariable analysis: *OR* 1.22; 95% CI 0.89–1.68; *P =* 0.223; multivariable analysis: *OR* 1.30; 95% CI 0.92–1.82; *P =* 0.138) (Table 2; Table S4) and ICU mortality (univariable analysis: *OR* 1.18; 95% CI 0.74–1.88; *P =* 0.479; multivariable analysis: *OR* 1.39; 95% CI 0.83–2.32, *P =* 0.206) (Table 2; Table S5). Likewise, the results of linear regression analysis revealed that oxygen therapy was associated with prolonged ICU LOS (*OR* 1.11; 95% CI 1.06–1.15; *P =* 0.009) and hospital LOS (*OR* 1.06; 95% CI 1.01–1.10; *P =* 0.009) (Table 2).

### Subgroupanalysis

A series of subgroupanalyses were performed to validate the robustness of our findings (Table 3). When taking the SpO_2_ target of oxygen therapy into consideration, we noticed that patients with a high-normal range of 95% < SpO_2_ ≤ 100% were associated with an increased risk of in-hospital mortality when compared with the ambient-air group (*OR* 1.44; 95% CI 1.03–2.03, *P =* 0.034). Oxygen therapy was also associated with deteriorative in-hospital mortality in AHF patients receiving oxygen therapy for > 6 days (*OR* 3.34; 95% CI 2.15–5.20; *P* <  0.001), and the history of atrial fibrillation (*OR* 1.69; 95% CI 1.00–2.87; *P =* 0.049). Other subgroups were not found to be significant.

**Table 3:** The association between oxygen therapy and all-cause in-hospital mortality in subgroup analysis

Characteristics	No. of total	No. Ambient Air (No. of deaths)	No. Oxygen Therapy (No. of deaths)	***OR*** (95% CI)	***P*** value
Duration of oxygen therapy (days)					
≤ 3	741	–	741 (40)	0.87 (0.60–1.27)	0.472
3–6	322	–	322 (28)	1.45 (0.94–2.25)	0.092
> 6	167	–	167 (30)	3.34 (2.15–5.20)	< 0.001
Median SpO_2_ during treatment period (%)					
90–95	753	431 (33)	322 (26)	1.06 (0.62–1.81)	0.832
> 95	2169	1261 (71)	908 (72)	1.44 (1.03–2.03)	0.034
Age (years)					
≤ 72	1498	856 (41)	642 (38)	1.25 (0.79–1.97)	0.334
> 72	1424	836 (63)	588 (60)	1.39 (0.96–2.02)	0.079
de novo AHF (*n*)					
Yes	1155	659 (58)	496 (58)	1.37 (0.92–2.22)	0.111
No	1767	1033 (46)	734 (40)	1.24 (0.85–1.82)	0.258
AF (*n*)					
Yes	668	381 (28)	287 (34)	1.69 (1.00–2.87)	0.049
No	2254	1311 (76)	943 (64)	1.18 (0.84–1.67)	0.337
MI (*n*)					
Yes	561	331 (33)	230 (22)	0.96 (0.54–1.69)	0.874
No	2361	1361 (71)	1000 (76)	1.49 (1.07–2.09)	0.019
Stroke (*n*)					
Yes	301	178 (14)	123 (17)	1.88 (0.89–3.97)	0.099
No	2621	1514 (90)	1107 (81)	1.25 (0.92–1.70)	0.161
Hypertension (*n*)					
Yes	1869	1041 (61)	828 (59)	1.23 (0.85–1.79)	0.268
No	1053	651 (43)	402 (39)	1.52 (0.97–2.39)	0.070
CKD (*n*)					
Yes	775	451 (40)	324 (36)	1.28 (0.80–2.07)	0302
No	2147	1241 (64)	906 (62)	1.35 (0.94–1.94)	0.102
RRT (*n*)					
Yes	149	102 (4)	47 (6)	3.59 (0.96–13.38)	0.057
No	2773	1590 (100)	1183 (92)	1.26 (0.94–1.69)	0.128

*OR* odds ratio, *CI* confidence interval, *SpO*_*2*_ pulse oximetry-derived oxygen saturation, *AHF* acute heart failure, *AF* atrial fibrillation, *MI* myocardial infarction, *CKD* chronic kidney disease, *RRT* renal replacement treatment, *−* no data

## Discussion

This study demonstrated that routine oxygen therapy was not found to reduce the composite endpoints of all-cause in-hospital and ICU mortality rates in normoxemic patients with AHF. The results also suggested that oxygen therapy might be closely correlated to increased LOS in ICU and hospital. Since the competitive risks existed in the secondary endpoints of ICU and hospital LOS, the significant correlation of oxygen supplementation with prolonged ICU ad hospital LOS should be interpreted cautiously. In addition, the absence of an effect of supplemental oxygen on mortality was consistent in all subgroups. To our knowledge, this study was the first to investigate the association between supplemental oxygen and short-term clinical outcomes based on a multi-center and critical care cohort of AHF patients without hypoxia.

Oxygen therapy remains a cornerstone for the treatment of AHF, while guidelines provide various recommendations on its appropriate use, reflecting the lack of robust evidence on such “cornerstone” therapy. The Canadian Cardiovascular Society (CCS) HF guidelines have recommended that supplemental oxygen should be considered for patients who are hypoxemic, and should be used cautiously in normoxemic patients due to concerns of increasing systemic vascular resistance and reducing cardiac output [27]. The latest European Society of Cardiology (ESC) and the Taiwan Society of Cardiology (TSOC) guidelines have indicated that oxygen should not be used routinely for AHF patients without hypoxemia [2, 28]. Recommendations on oxygen therapy in normoxemic patients with AHF are not mentioned in the latest American College of Cardiology (ACC)/American Heart Association (AHA)/Heart Failure Society of America (HFSA) [1], or the National Institute for Health and Care Excellence (NICE) guidelines [16]. However, most of these recommendations appear to be solely based on expert opinion rather than high-quality evidence, and we hope our findings might provide certain evidence for this point of view. In addition, one observational study documented supplemental oxygen to be prescribed among at least half of AHF patients in the emergency department (ED), regardless of SpO_2_ level [4]. A lack of clinical benefit could mean that by departing from this practice, it might be significant not only for patients to have a lower treatment burden but also to have reduced medical expenses.

Our findings were consistent with the results of studies that evaluated the impacts of supplemental oxygen therapy in other clinical settings. Several RCTs and meta-analyses have demonstrated that oxygen therapy does not significantly reduce all-cause mortality, and can even increase the incidence of early myocardial injury and infarct size among AMI patients with normoxemia [8, 10–13, 29–33]. A total of 11 RCTs including 6366 patients with acute stroke showed a nonsignificant increase in mortality at 3, 6, and 12 months in patients who received normobaric oxygen compared with those who received ambient air [34]. In addition, it was standard to perform neonatal resuscitation with 100% oxygen until multiple RCTs demonstrated that room air resulted in a lower incidence of infant mortality and hypoxic ischemic encephalopathy than 100% oxygen, thereby contributing to a dramatic change in guidelines and practice [35].

Although hypoxic patients can benefit from supplemental oxygen to correct hypoxia, supplementation above normoxia seems to be futile as the hemodynamic response to hyperoxia outbalances the benefit of additionally dissolved oxygen in the blood. The cardiovascular effects of hyperoxia are mainly mediated by following pathways: endothelial production of ROS [36] and hyperoxia-induced vasoconstriction in the coronary [37], retinal [38], and cerebral vascular bed [39]. The excessive formation of ROS outweighs the antioxidant capacities of the cells, and results in oxidative stress and a cascade of adverse outcomes including damage to nucleic acids, proteins and lipids, and activation of apoptotic as well as necrotic pathways causing cell death [6, 40, 41]. Furthermore, ROS-induced closure of adenosine triphosphate (ATP)-dependent potassium channels and activation of ligand-gated calcium channels in vascular smooth muscle cells lead to the peripheral vasoconstriction and decreased regional blood flow in most vascular beds [42, 43]. Ruggiu et al. [44] demonstrated that hyperoxia at any time of the ICU stay significantly decreases OS and is an independent mortality risk factor regardless of the cause of patient admission. A study showed that extreme hyperoxia [FiO_2_ = 1.0; arterial partial pressure of oxygen (PaO_2_) = 300 mmHg] was associated with an 8 to 30% decrease in coronary blood flow, impairment of cardiac relaxation and contractility, and increased left ventricular filling pressures in patients with congestive HF [45]. In addition, two studies revealed that hyperoxia caused peripheral vasoconstriction, increased systemic vascular resistance, and did not increase systemic oxygen delivery in HF patients [19, 46]. Haque et al. found an increase in pulmonary capillary wedge pressure caused by hyperoxia in AHF patients with this effect starting at an FiO_2_ level of 0.24 [47]. However, Nael et al. [48] conducted a study to evaluate the impact of early hyperoxia exposure among AHF patients admitted with pulmonary congestion and treated with oxygen therapy, and did not find any difference in 30-day mortality between patients with phases of hyperoxia and without hyperoxia. Therefore, a multicenter, prospective, RCT is needed to further assess the association between hyperoxia and mortality in AHF patients, and provide a definitive answer about the consequences.

The goal of oxygen therapy remains uncertain for AHF patients. In our subgroup analysis, we divided patients by mean SpO_2_ level during hospitalization into a low-normal (90% ≤ SpO_2_ ≤ 95%) and a high-normal (95% < SpO_2_ ≤ 100%) cohort. Among patients with a high-normal range of 95% < SpO_2_ ≤ 100%, oxygen therapy was associated with worse clinical outcomes compared to the ambient-air group. Although measurement of SpO_2_ might be inappropriate tool to quantify the magnitude of hyperoxemia, some researchers reported that the prevalence of arterial hyperoxia increased when SpO_2_ was > 95% in a cross-sectional study of 100 mechanically ventilated patients admitted to the ICU [49]. Improving Oxygen Therapy in Acute-illness (IOTA) systematic review and meta-analysis concluded that liberal oxygen therapy (without setting an upper limit of SpO_2_) could increase mortality, and SpO_2_ should not exceed 96% among acutely ill adults [50]. Additionally, the Thoracic Society of Australia and New Zealand (TSANZ) and the British Thoracic Society (BTS) have recommended that oxygen is stopped above an upper limit of SpO_2_ in critically ill patients (TSANZ: 96%; BTS: 98%) [51, 52]. However, a recent study investigated the effect of oxygen titrated to high (SpO_2_ ≥ 96%) vs. low (90% ≤ SpO_2_ ≤ 92%) pulse oximetry targets in 50 patients hospitalized with AHF, and implicated that the difference of baseline and 72 h N-terminal pro-brain-type natriuretic peptide (NT-proBNP) levels were of no obvious significance between groups with high and low SpO_2_ targets [53]. Nonetheless, limited by the small sample size and few death events, they did not assess the impact of these two groups on clinical endpoints (such as in-hospital mortality).

Notablely, the current study must be interpreted in the context of several limitations. First, all patients with an admission SpO_2_ level ≥ 90% were included in our study cohort, yet misclassification could not be ruled out, as SpO_2_ was measured by local procedures and therefore subjected to selection bias. Second, to avoid including patients developing refractory and progressive hypoxemia during intervention, we excluded patients receiving more intensive oxygen therapy including endotracheal intubation and NIV during hospitalization. However, this might lead to selection biases, and these patients receiving more advanced oxygen support should be considered separately in further study. Third, the eICU database did not provide any information before patients’ admission, therefore we could not identify whether patients enrolled in this study received oxygen therapy prior to admission. Fourth, we did not include detailed information with regard to oxygen therapy (oxygen flow rate and oxygen concentration), or some important laboratory variables (such as NT-proBNP) due to more than 20% missing values, which might be related to the efficacy of oxygen therapy. Another limitation was the retrospective nature of the study. Retrospective studies almost always have bias because prognostic factors are unequally distributed between patients exposed or not exposed to an intervention. Although multivariate logistic regression and PSM analysis were applied in the present study to control the covariate imbalance and selection bias, more high-quality clinical trials are needed to strengthen our results.

## Conclusions

Routine use of supplemental oxygen in normoxemic patients with AHF was not found to reduce the composite of all-cause in-hospital or ICU mortality. Thus, our results provided supporting evidence for the rationale use of oxygen therapy in the current guidelines.

## Supplementary Information

**Additional file 1.**

## Data Availability

The datasets generated and/ or analyzed during the current study are available from the corresponding author upon reasonable request.

## Abbreviations

ACC: American College of Cardiology
ACEI/ARB: Angiotensin-converting-enzyme inhibitors/angiotensin receptor blockers
AHA: American Heart Association
AHF: Acute heart failure
AMI: Acute myocardial infarction
ATP: Adenosine triphosphate
BMI: Body mass index
BTS: British Thoracic Society
CAS: Coronary artery disease
CCB: Calcium channel blocker
CCS: Canadian Cardiovascular Society
CI: Confidence interval
ED: Emergency department
eICU: Electronic Intensive Care Unit
ESC: European Society of Cardiology
FiO_2_: Fraction of inspired oxygen
GCS: Glasgow Coma Scale
HF: Heart failure
HFSA: Heart Failure Society of America
ICD-9: International Classification of Diseases, Ninth Revision
IOTA: Improving Oxygen Therapy in Acute-illness
IQR: Interquartile range
LOS: Length of stay
NICE: National Institute for Health and Care Excellence
NIV: Non-invasive ventilation
NT-proBNP: N-terminal pro-brain-type natriuretic peptide
OASIS: Oxford Acute Severity of Illness Score
OR: Odds ratio
PaO_2_: Arterial partial pressure of oxygen
PSM: Propensity score matching
RCT: Randomized controlled trial
ROS: Reactive oxygen species
SD: Standardized difference
SMD: Standardized mean difference
SOFA: Sequential Organ Failure Assessment
SpO_2_: Pulse oximetry-derived oxygen saturation
SQL: Structured query language
STROBE: STrengthening the Reporting of OBservational studies in Epidemiology
TSANZ: Thoracic Society of Australia and New Zealand
TSOC: Taiwan Society of Cardiology
