# Supplementary material for: Is oxygen therapy beneficial for normoxemic patients with acute heart failure? A propensity score matched study
Source: Mil Med Res. 2021 Jul 9;8:38. doi: 10.1186/s40779-021-00330-7 (PMC8268364; doi:10.1186/s40779-021-00330-7)
Supplement: Supplementary file 1 — Additional file 1. [file 40779_2021_330_MOESM1_ESM.docx]

# Additional files

**Table S1** Univariable and multivariable logistic regression analysis for all-cause in-hospital mortality before matching

| **Characteristics** | **Univariable**  **OR (95% CI)** | ***P* value** | **Multivariable**  **OR (95% CI)** | ***P* value** |
| --- | --- | --- | --- | --- |
| Demographics |  |  |  |  |
| Age | 1.02 (1.01 - 1.03) | < 0.001 | 1.03 (1.01 - 1.04) | < 0.001 |
| Gender |  |  |  |  |
| Male | 1 | - | - | - |
| Female | 1.04 (0.78 - 1.38) | 0.810 | - | - |
| BMI | 1.00 (1.00 - 1.01) | 0.365 | - | - |
| *de novo* AHF | 0.87 (0.65 - 1.17) | 0.350 |  |  |
| Comorbidities on admission |  |  |  |  |
| Sepsis | 1.89 (1.2 - 2.78) | 0.001 | 1.06 (0.69 - 1.64) | 0.791 |
| AKI | 1.13 (0.45 - 2.83) | 0.802 |  |  |
| History of disease |  |  |  |  |
| AF | 1.5 (1.13 - 2.11) | 0.006 | 1.14 (0.81 - 1.61) | 0.451 |
| CAD |  |  |  |  |
| MI | 1.64 (1.18 - 2.27) | 0.003 | 1.56 (1.10 - 2.21) | 0.013 |
| PCI | 1.21 (0.79 - 1.84) | 0.380 |  |  |
| CABG | 0.86 (0.54 - 1.37) | 0.530 |  |  |
| Hypertension | 0.81 (0.61 - 1.09) | 0.163 |  |  |
| Stroke | 1.65 (1.10 - 2.46) | 0.015 | 1.52 (0.98 - 2.35) | 0.061 |
| DM | 1.00 (0.74 - 1.34) | 0.982 |  |  |
| CKD | 1.74 (1.30 - 2.34) | < 0.001 | 1.38 (0.97 - 1.95) | 0.071 |
| Hyperthyroidism | 1.33 (0.89 - 1.99) | 0.171 |  |  |
| Vital signs at presentation |  |  |  |  |
| SBP | 0.95 (0.93 - 0.97) | < 0.001 | 0.98 (0.96 - 1.00) | 0.025 |
| HR | 1.01 (1.00 - 1.01) | 0.156 | - | - |
| SpO2 | 1.01 (0.96 - 1.07) | 0.689 | - | - |
| Laboratory findings and blood gas analysis |  |  |  |  |
| Albumin | 0.60 (0.48 - 0.76) | < 0.001 | 0.73 (0.56 - 0.96) | 0.023 |
| Creatinine | 1.07 (1.00 - 1.15) | 0.045 | 0.88 (0.77 - 1.00) | 0.048 |
| Glucose | 1.00 (1.00 - 1.00) | 0.468 |  |  |
| BUN | 1.01 (1.01 - 1.02) | < 0.001 | 1.01 (1.00 - 1.01) | 0.158 |
| Hematocrit | 0.98 (0.96 - 1.01) | 0.137 |  |  |
| Hemoglobin | 0.93 (0.88 - 0.99) | 0.031 | 1.02 (0.95 - 1.09) | 0.667 |
| Platelet | 1.00 (1.00 - 1.00) | 0.012 | 1.00 (1.00 - 1.00) | 0.403 |
| WBC | 1.02 (1.01 - 1.03) | 0.006 | 1.01 (1.00 - 1.03) | 0.017 |
| Potassium | 1.43 (1.13 - 1.80) | 0.003 | 1.35 (1.03 - 1.77) | 0.028 |
| Sodium | 0.98 (0.96 - 1.01) | 0.220 |  |  |
| Scoring system |  |  |  |  |
| OASIS | 1.04 (1.02 - 1.05) | < 0.001 | 1.00 (0.98 - 1.03) | 0.790 |
| SOFA | 1.34 (1.27 - 1.41) | < 0.001 | 1.29 (1.19 - 1.40) | <0.001 |
| GCS | 0.70 (0.59 - 0.83) | < 0.001 | 0.91 (0.75 - 1.11) | 0.370 |
| Management of AHF |  |  |  |  |
| Oxygen therapy | 1.32 (0.99 - 1.76) | 0.056 | 1.34 (0.98 - 1.84) | 0.067 |
| Intra-aortic balloon pump | 1.06 (0.33 - 3.48) | 0.918 |  |  |
| RRT | 0.97 (0.50 - 1.87) | 0.921 |  |  |
| In-hospital medication |  |  |  |  |
| Inotropes | 2.46 (1.57 - 3.85) | < 0.001 | 1.13 (0.68 - 1.89) | 0.642 |
| Diuretic | 2.31 (1.46 - 3.65) | < 0.001 | 1.52 (0.91 - 2.56) | 0.114 |
| ACEI/ARB | 0.50 (0.26 - 0.95) | 0.033 | 0.81 (0.40 - 1.65) | 0.560 |
| CCB | 1.02 (0.56 - 1.86) | 0.956 | - | - |
| Beta-blocker | 0.65 (0.45 - 0.95) | 0.026 | 0.78 (0.51 - 1.19) | 0.251 |

*OR* odds ratio, *CI* confidence interval, *BMI* body mass index, *AHF* acute heart failure, *AKI* acute kidney injury, *AF* atrial fibrillation, *CAD* coronary artery disease, *MI* myocardial infarction, *PCI* percutaneous coronary intervention, *CABG* coronary artery bypass grafting, *DM* diabetes mellitus, *CKD* chronic kidney disease, *SBP* systolic blood pressure, *HR* heart rate, *SpO_2_* pulse oximetry-derived oxygen saturation, *BUN* blood urea nitrogen, *WBC* white blood cell, *OASIS* oxford acute severity of illness score, *SOFA* sequential organ failure assessment score, *GCS* glasgow coma scale, *RRT* renal replacement treatment, *ACEI/ARB* angiotensin-converting-enzyme inhibitors/angiotensin receptor blockers, *CCB* calcium channel blocker, - no data.

**Table S2** Univariable and multivariable logistic regression analysis for all-cause ICU mortality before matching

| **Characteristics** | **Univariable**  ***OR* (95% CI)** | ***P* value** | **Multivariable**  ***OR* (95% CI)** | ***P* value** |
| --- | --- | --- | --- | --- |
| Demographics |  |  |  |  |
| Age | 1.02 (1.00 - 1.04) | 0.017 | 1.03 (1.01, 1.05) | 0.001 |
| Gender |  |  |  |  |
| Male | 1 | - | - | - |
| Female | 1.22 (0.79 - 1.88) | 0.362 | - | - |
| BMI | 1.00 (0.98 - 1.02) | 0.741 | - | - |
| *de novo* AHF | 0.89 (0.57 - 1.37) | 0.589 |  |  |
| Comorbidities on admission |  |  |  |  |
| Sepsis | 1.57 (0.86 - 2.87) | 0.144 | - | - |
| AKI | 1.64 (0.50 - 5.33) | 0.412 | - | - |
| History of disease |  |  |  |  |
| AF | 1.26 (0.78 - 2.05) | 0.351 | - | - |
| CAD |  |  |  |  |
| MI | 1.31 (0.78 - 2.17) | 0.305 | - | - |
| PCI | 0.80 (0.38 - 1.67) | 0.547 | - | - |
| CABG | 0.36 (0.13 - 1.00) | 0.049 | 0.30 (0.10, 0.85) | 0.024 |
| Hypertension | 0.84 (0.54 - 1.31) | 0.440 | - | - |
| Stroke | 1.75 (0.98 - 3.15) | 0.061 | - | - |
| DM | 0.94 (0.60 - 1.46) | 0.768 | - | - |
| CKD | 1.16 (0.72 - 1.86) | 0.541 | - | - |
| Hyperthyroidism | 1.62 (0.92 - 2.87) | 0.096 | - | - |
| Vital signs at presentation |  |  |  |  |
| SBP | 0.95 (0.93 - 0.98) | 0.001 | 0.98 (0.95, 1.02) | 0.309 |
| HR | 1.00 (0.99 - 1.01) | 0.863 | - | - |
| SpO2 | 0.99 (0.92 - 1.07) | 0.821 | - | - |
| Laboratory findings and blood gas analysis |  |  |  |  |
| Albumin | 0.66 (0.47 - 0.94) | 0.020 | 0.80 (0.54, 1.19) | 0.267 |
| Creatinine | 1.03 (0.92 - 1.15) | 0.653 |  |  |
| Glucose | 0.99 (0.99 - 0.99) | 0.023 | 0.99 (0.99, 1.00) | 0.043 |
| BUN | 1.02 (1.01 - 1.02) | < 0.001 | 1.00 (0.99, 1.01) | 0.539 |
| Hematocrit | 0.98 (0.95 - 1.01) | 0.187 | - | - |
| Hemoglobin | 0.92 (0.83 - 1.01) | 0.069 | - | - |
| Platelet | 1.00 (1.00 - 1.00) | 0.096 | - | - |
| WBC | 1.02 (1.01 - 1.04) | 0.003 | 1.02 (1.01, 1.03) | 0.006 |
| Potassium | 1.64 (1.16 - 2.31) | 0.005 | 1.37 (0.92, 2.05) | 0.123 |
| Sodium | 1.00 (0.96 - 1.04) | 0.865 | - | - |
| Scoring system |  |  |  |  |
| OASIS | 1.04 (1.01 - 1.06) | 0.005 | 1.00 (0.97, 1.04) | 0.885 |
| SOFA | 1.42 (1.32 - 1.53) | < 0.001 | 1.36 (1.22, 1.51) | < 0.001 |
| GCS | 0.67 (0.53 - 0.85) | 0.001 | 0.82 (0.61, 1.09) | 0.169 |
| Management of AHF |  |  |  |  |
| Oxygen therapy | 1.42 (0.92 - 2.19) | 0.109 | 1.58 (0.97, 2.56) | 0.066 |
| Intra-aortic balloon pump | 0 | 0.982 | - | - |
| RRT | 0.44 (0.11 - 1.81) | 0.256 | - | - |
| In-hospital medication |  |  |  |  |
| Inotropes | 3.02 (1.64 - 5.57) | < 0.001 | 1.14 (0.55, 2.35) | 0.731 |
| Diuretic | 3.00 (1.63 - 5.53) | 0.043 | 1.76 (0.87, 3.57) | 0.119 |
| ACEI/ARB | 0.74 (0.32 - 1.72) | 0.489 | - | - |
| CCB | 0.38 (0.09 - 1.56) | 0.180 | - | - |
| Beta-blocker | 0.52 (0.28 - 0.96) | 0.037 | 0.64 (0.32, 1.26) | 0.194 |

*ICU* intensive care unit, *OR* odds ratio, *CI* confidence interval, *BMI* body mass index, *AHF* acute heart failure, *AKI* acute kidney injury, *AF* atrial fibrillation, *CAD* coronary artery disease, *MI* myocardial infarction, *PCI* percutaneous coronary intervention, *CABG* coronary artery bypass grafting, *DM* diabetes mellitus, *CKD* chronic kidney disease, *SBP* systolic blood pressure, *HR* heart rate, *SpO_2_* pulse oximetry-derived oxygen saturation, *BUN* blood urea nitrogen, *WBC* white blood cell, *OASIS* oxford acute severity of illness score, *SOFA* sequential organ failure assessment score, *GCS* glasgow coma scale, *RRT* renal replacement treatment, *ACEI/ARB* angiotensin-converting-enzyme inhibitors/angiotensin receptor blockers, *CCB* calcium channel blocker, - no data.

**Table S3** Comparisons after propensity score matching

| **Characteristics** | **Ambient Air**  ***n =* 1122** | **Oxygen**  ***n =* 1122** | **SMD** | ***P* value** |
| --- | --- | --- | --- | --- |
| Demographics |  |  |  |  |
| Age (years) | 72 (61-82) | 72 (60-83) | 0.023 | 0.847 |
| Gender [male, *n* (%)] | 604 (53.8) | 610 (54.4) | 0.011 | 0.799 |
| BMI (kg/m^2^) | 28.4 (24.4-34.0) | 28.6 (24.1-34.7) | 0.018 | 0.941 |
| *de novo* AHF [*n* (%)] | 439 (39.1) | 448 (39.9) | 0.016 | 0.698 |
| Comorbidities on admission [*n* (%)] |  |  |  |  |
| Sepsis | 8 (10.5) | 113 (10.1) | 0.015 | 0.728 |
| AKI | 23 (2.1) | 19 (1.7) | 0.026 | 0.533 |
| History of disease [*n* (%)] |  |  |  |  |
| AF | 269 (24.0) | 264 (23.5) | 0.010 | 0.804 |
| CAD |  |  |  |  |
| MI | 216 (19.3) | 211 (18.8) | 0.011 | 0.788 |
| PCI | 132 (11.8) | 128 (11.4) | 0.011 | 0.792 |
| CABG | 139 (12.4) | 133 (11.9) | 0.016 | 0.698 |
| Hypertension | 735 (65.5) | 739 (65.9) | 0.008 | 0.859 |
| Stroke | 115 (10.3) | 115 (10.3) | <0.001 | 0.998 |
| DM | 446 (39.8) | 447 (39.8) | 0.002 | 0.966 |
| CKD | 288 (25.7) | 290 (25.8) | 0.004 | 0.923 |
| Hyperthyroidism | 144 (12.8) | 138 (12.3) | 0.016 | 0.702 |
| Vital signs at presentation |  |  |  |  |
| SBP (mmHg) | 121.0 (120.0-127.0) | 121.0 (120.0-127.0) | 0.004 | 0.848 |
| HR (beats/min) | 86.0 (72.0-102.0) | 86.0 (74.0-101.0) | 0.014 | 0.558 |
| SpO_2_ (%) | 97.0 (95.0-99.0) | 97.0 (95.0-99.0) | 0.002 | 0.624 |
| Laboratory findings and blood gas analysis |  |  |  |  |
| Albumin (mg/dL) | 3.1 (2.7-3.5) | 3.1 (2.6-3.5) | 0.007 | 0.853 |
| Creatinine (μmol/L) | 1.3 (1.0-2.0) | 1.3 (0.9-2.1) | 0.010 | 0.643 |
| Glucose (mg/dL) | 109.0 (92.0-135.8) | 109.5 (91.0-139.0) | 0.006 | 0.760 |
| BUN (mg/dL) | 26.0 (18.0-43.0) | 27.0 (18.0-44.0) | 0.027 | 0.296 |
| Hematocrit (%) | 32.2 (6.9) | 32.2 (6.8) | 0.004 | 0.837 |
| Hemoglobin (g/dL) | 10.5 (8.8-12.1) | 10.4 (8.9-12.1) | 0.008 | 0.908 |
| Platelet (10^9^ /L) | 183.0 (135.0-243.0) | 185.0 (137.0-241.7) | 0.017 | 0.908 |
| WBC (10^9^/L) | 8.5 (6.3-11.4) | 8.5 (6.3-11.7) | 0.008 | 0.808 |
| Potassium (mmol/L) | 3.9 (3.5-4.3) | 3.9 (3.5-4.3) | 0.017 | 0.610 |
| Sodium (mmol/L) | 136.0 (133.0-139.0) | 136.0 (133.0-139.0) | 0.002 | 0.770 |
| Scoring system |  |  |  |  |
| OASIS | 19.0 (13.0-24.0) | 19.0 (12.0-25.0) | 0.013 | 0.800 |
| SOFA | 4.0 (2.0-6.0) | 4.0 (2.0-6.0) | 0.021 | 0.676 |
| GCS | 15.0 (15.0-15.0) | 15.0 (14.3-15.0) | <0.001 | 0.600 |
| Management of AHF [*n* (%)] |  |  |  |  |
| Intra-aortic balloon pump | 17 (1.6) | 15 (1.3) | 0.015 | 0.722 |
| RRT | 46 (4.1) | 47 (4.2) | 0.004 | 0.916 |
| In-hospital medication [*n* (%)] |  |  |  |  |
| Inotropes | 183 (16.3) | 192 (17.1) | 0.039 | 0.351 |
| Diuretic | 855 (76.2) | 862 (76.8) | 0.025 | 0.548 |
| ACEI/ARB | 665 (59.3) | 681 (60.7) | 0.048 | 0.260 |
| CCB | 70 (6.2) | 72 (6.4) | 0.011 | 0.794 |
| Beta-blocker | 619 (55.2) | 661 (58.9) | 0.082 | 0.051 |

*Values are *n* (%) or median (interquartile range). *SMD* standardized mean difference, *BMI* body mass index, *AHF* acute heart failure, *AKI* acute kidney injury, *AF* atrial fibrillation, *CAD* coronary artery disease, *MI* myocardial infarction, *PCI* percutaneous coronary intervention, *CABG* coronary artery bypass grafting, *DM* diabetes mellitus, *CKD* chronic kidney disease, *SBP* systolic blood pressure, *HR* heart rate, *SpO_2_* pulse oximetry-derived oxygen saturation, *BUN* blood urea nitrogen, *WBC* white blood cell, *OASIS* oxford acute severity of illness score, *SOFA* sequential organ failure assessment score, *GCS* glasgow coma scale, *RRT* renal replacement treatment, *ACEI/ARB* angiotensin-converting-enzyme inhibitors/angiotensin receptor blockers, *CCB* calcium channel blocker.

**Table S4** Univariable and multivariable logistic regression analysis for all-cause in-hospital mortality after matching

| **Characteristics** | **Univariable**  ***OR* (95% CI)** | ***P* value** | **Multivariable**  ***OR* (95% CI)** | ***P* value** |
| --- | --- | --- | --- | --- |
| Demographics |  |  |  |  |
| Age | 1.02 (1.01 - 1.04) | 0.001 | 1.03 (1.01 - 1.04) | <0.001 |
| Gender |  |  |  |  |
| Male | 1 | - | - | - |
| Female | 1.09 (0.79 - 1.50) | 0.604 | - | - |
| BMI | 1.00 (0.99 - 1.01) | 0.502 | - | - |
| *de novo* AHF |  |  |  |  |
| Comorbidities on admission |  |  |  |  |
| Sepsis | 2.21 (1.46 - 3.35) | < 0.001 | 1.14 (0.71 - 1.83) | 0.592 |
| AKI | 1.35 (0.48 - 3.84) | 0.570 | - | - |
| History of disease |  |  |  |  |
| AF | 1.46 (1.03 - 2.07) | 0.032 | 1.11 (0.76 - 1.64) | 0.586 |
| CAD |  |  |  |  |
| MI | 1.75 (1.23 - 2.51) | 0.002 | 1.80 (1.22 - 2.65) | 0.003 |
| PCI | 1.14 (0.71 - 1.84) | 0.591 | - | - |
| CABG | 0.66 (0.38 - 1.17) | 0.154 | - | - |
| Hypertension |  |  |  |  |
| Stroke | 1.57 (1.00 - 2.48) | 0.052 | - | - |
| DM | 1.15 (0.83 - 1.59) | 0.394 | - | - |
| CKD | 1.81 (1.30 - 2.52) | 0.001 | 1.27 (0.87 - 1.86) | 0.216 |
| Hyperthyroidism | 1.22 (0.78 - 1.92) | 0.389 | - | - |
| Vital signs at presentation |  |  |  |  |
| SBP | 0.94 (0.92 - 0.96) | < 0.001 | 0.97 (0.94 - 0.99) | 0.004 |
| HR | 1.01 (1.00 - 1.02) | 0.060 | - | - |
| SpO_2_ | 1.02 (0.97 - 1.09) | 0.433 | - | - |
| Laboratory findings and blood gas analysis |  |  |  |  |
| Albumin | 0.57 (0.44 - 0.73) | < 0.001 | 0.74 (0.55 - 0.99) | 0.040 |
| Creatinine | 1.06 (0.98 - 1.16) | 0.144 | - | - |
| Glucose | 1.00 (1.00 - 1.00) | 0.524 | - | - |
| BUN | 1.02 (1.01 - 1.02) | < 0.001 | 1.00 (0.99 - 1.01) | 0.905 |
| Hematocrit | 0.99 (0.96 - 1.01) | 0.190 |  |  |
| Hemoglobin | 0.93 (0.87 - 1.00) | 0.053 |  |  |
| Platelet | 1.00 (1.00 - 1.00) | 0.022 | 1.00 (1.00 - 1.00) | 0.435 |
| WBC | 1.02 (1.00 - 1.03) | 0.016 | 1.01 (1.00 - 1.02) | 0.051 |
| Potassium | 1.51 (1.16 - 1.96) | 0.002 | 1.39 (1.02 - 1.89) | 0.038 |
| Sodium | 0.98 (0.95 - 1.01) | 0.124 |  |  |
| Scoring system |  |  |  |  |
| OASIS | 1.04 (1.02 - 1.06) | < 0.001 | 0.99 (0.97 - 1.01) | 0.420 |
| SOFA | 1.35 (1.28 - 1.43) | < 0.001 | 1.27 (1.16 - 1.38) | <0.001 |
| GCS | 0.67 (0.55 - 0.80) | < 0.001 | 0.86 (0.69 - 1.06) | 0.157 |
| Management of AHF |  |  |  |  |
| Oxygen therapy | 1.22 (0.89 - 1.68) | 0.223 | 1.30 (0.92 - 1.82) | 0.138 |
| Intra-aortic balloon pump | 0.85 (0.20 - 3.59) | 0.824 | - | - |
| RRT | 1.21 (0.58 - 2.55) | 0.612 | - | - |
| In-hospital medication |  |  |  |  |
| Inotropes | 2.40 (1.48 - 3.89) | < 0.001 | 1.22 (0.70 - 2.10) | 0.482 |
| Diuretic | 2.33 (1.43 - 3.82) | 0.001 | 1.70 (0.97 - 2.98) | 0.062 |
| ACEI/ARB | 0.57 (0.30 - 1.10) | 0.093 | - | - |
| CCB | 0.86 (0.43 - 1.73) | 0.678 | - | - |
| Beta-blocker | 0.59 (0.39 - 0.88) | 0.011 | 0.76 (0.49 - 1.18) | 0.214 |

*OR* odds ratio, *CI* confidence interval, *BMI* body mass index, *AHF* acute heart failure, *AKI* acute kidney injury, *AF* atrial fibrillation, *CAD* coronary artery disease, *MI* myocardial infarction, *PCI* percutaneous coronary intervention, *CABG* coronary artery bypass grafting, *DM* diabetes mellitus, *CKD* chronic kidney disease, *SBP* systolic blood pressure, *HR* heart rate, *SpO_2_* pulse oximetry-derived oxygen saturation, *BUN* blood urea nitrogen, *WBC* white blood cell, *OASIS* oxford acute severity of illness score, *SOFA* sequential organ failure assessment score, *GCS* glasgow coma scale, *RRT* renal replacement treatment, *ACEI/ARB* angiotensin-converting-enzyme inhibitors/angiotensin receptor blockers, *CCB* calcium channel blocker, - no data.

**Table S5** Univariable and multivariable logistic regression analysis for all-cause ICU mortality after matching

| **Characteristics** | **Univariable**  ***OR* (95% CI)** | ***P* value** | **Multivariable**  ***OR* (95% CI)** | ***P* value** |
| --- | --- | --- | --- | --- |
| Demographics |  |  |  |  |
| Age | 1.02 (1.01 - 1.04) | 0.011 | 1.04 (1.02 - 1.06) | 0.001 |
| Gender |  |  |  |  |
| Male | 1 | - | - | - |
| Female | 1.25 (0.79 - 1.99) | 0.340 | - | - |
| BMI | 0.99 (0.97 - 1.02) | 0.556 | - | - |
| *de novo* AHF | 0.81 (0.51 - 1.29) | 0.366 | - | - |
| Comorbidities on admission |  |  |  |  |
| Sepsis | 1.72 (0.92 - 3.25) | 0.091 | - | - |
| AKI | 2.31 (0.70 - 7.65) | 0.171 | - | - |
| History of disease |  |  |  |  |
| AF | 1.28 (0.77 - 2.15) | 0.343 | - | - |
| CAD |  |  |  |  |
| MI | 1.28 (0.74 - 2.22) | 0.381 | - | - |
| PCI | 0.54 (0.22 - 1.36) | 0.193 | - | - |
| CABG | 0.30 (0.09 - 0.96) | 0.042 | 0.25 (0.08 - 0.82) | 0.023 |
| Hypertension | 0.76 (0.47 - 1.22) | 0.253 | - | - |
| Stroke | 1.56 (0.81 - 3.00) | 0.187 | - | - |
| DM | 0.87 (0.54 - 1.40) | 0.555 | - | - |
| CKD | 1.15 (0.69 - 1.92) | 0.600 | - | - |
| Hyperthyroidism | 1.51 (0.82 - 2.78) | 0.190 | - | - |
| Vital signs at presentation |  |  |  |  |
| SBP | 0.95 (0.92 - 0.98) | < 0.001 | 0.97 (0.94 - 1.01) | 0.126 |
| HR | 1.00 (0.99 - 1.01) | 0.908 | - | - |
| SpO_2_ | 0.99 (0.91 - 1.08) | 0.879 | - | - |
| Laboratory findings and blood gas analysis |  |  |  |  |
| Albumin | 0.66 (0.46 - 0.96) | 0.031 | 0.97 (0.62 - 1.51) | 0.881 |
| Creatinine | 1.03 (0.90 - 1.17) | 0.684 | - | - |
| Glucose | 0.99 (0.99 - 1.00) | 0.012 | 0.99 (0.98 - 1.00) | 0.014 |
| BUN | 1.02 (1.01 - 1.02) | < 0.001 | 1.00 (0.99 - 1.02) | 0.470 |
| Hematocrit | 0.97 (0.94 - 1.01) | 0.115 | - | - |
| Hemoglobin | 0.90 (0.81 - 1.00) | 0.044 | 0.94 (0.83 - 1.06) | 0.287 |
| Platelet | 1.00 (1.00 - 1.00) | 0.039 | 1.00 (1.00 - 1.00) | 0.685 |
| WBC | 1.02 (1.01 - 1.03) | 0.007 | 1.02 (1.00 - 1.03) | 0.016 |
| Potassium | 1.56 (1.07 - 2.28) | 0.022 | 1.30 (0.83 - 2.05) | 0.256 |
| Sodium | 1.00 (0.96 - 1.05) | 0.998 | - | - |
| Scoring system |  |  |  |  |
| OASIS | 1.04 (1.01 - 1.07) | 0.008 | 0.99 (0.96 - 1.03) | 0.735 |
| SOFA | 1.41 (1.31 - 1.53) | < 0.001 | 1.36 (1.20 - 1.54) | <0.001 |
| GCS | 0.64 (0.50 - 0.83) | 0.001 | 0.76 (0.56 - 1.04) | 0.088 |
| Management of AHF |  |  |  |  |
| Oxygen therapy | 1.18 (0.74 - 1.88) | 0.479 | 1.39 (0.83 - 2.32) | 0.206 |
| Intra-aortic balloon pump | 0 | 0.984 | - | - |
| RRT | 0.64 (0.15 - 2.63) | 0.530 | - | - |
| In-hospital medication |  |  |  |  |
| Inotropes | 2.87 (1.51 - 5.46) | 0.001 | 1.10 (0.51 - 2.37) | 0.803 |
| Diuretic | 2.96 (1.56 - 5.64) | 0.001 | 1.76 (0.84 - 3.70) | 0.133 |
| ACEI/ARB | 0.79 (0.34 - 1.84) | 0.585 | - | - |
| CCB | 0.20 (0.03 - 1.44) | 0.110 | - | - |
| Beta-blocker | 0.41 (0.21 - 0.81) | 0.010 | 0.55 (0.26 - 1.15) | 0.110 |

*ICU* intensive care unit, *OR* odds ratio, *CI* confidence interval, *BMI* body mass index, *AHF* acute heart failure, *AKI* acute kidney injury, *AF* atrial fibrillation, *CAD* coronary artery disease, *MI* myocardial infarction, *PCI* percutaneous coronary intervention, *CABG* coronary artery bypass grafting, *DM* diabetes mellitus, *CKD* chronic kidney disease, *SBP* systolic blood pressure, *HR* heart rate, *SpO_2_* pulse oximetry-derived oxygen saturation, *BUN* blood urea nitrogen, *WBC* white blood cell, *OASIS* oxford acute severity of illness score, *SOFA* sequential organ failure assessment score, *GCS* glasgow coma scale, *RRT* renal replacement treatment, *ACEI/ARB* angiotensin-converting-enzyme inhibitors/angiotensin receptor blockers, *CCB* calcium channel blocker, - no data.
